# Celiac Disease and Viral B Hepatitis: Lessons for Clinical Practice

**Published:** 2010-12-01

**Authors:** Antonio Tursi

**Affiliations:** 1 Gastroenterology Service, ASL BAT, Andria (BAT), Italy

**Keywords:** Hepatitis B virus, Celiac disease

Dear Editor,

Celiac disease (CD) is a chronic inflammatory disease of the gut occurring in genetically susceptible individuals after the ingestion of gluten. It is characterized by a flattened mucosa, villous atrophy, and crypt hyperplasia in the small intestine, by the classic malabsorption syndrome (diarrhea, steatorrhea, weight loss), or by seemingly less severe symptoms such as iron deficiency anemia, osteopenic bone disease, amenorrhea, and infertility [1]. The elimination of gluten from the diet generally leads to a return to normality of the morphological changes [2]. Intestinal damage is caused by an interaction between the deamidated glutamine residues of gliadin and HLA-DQ2 (DQA1*05/DQB1*2) or DQ8 (DQA1*03/DQB1*0302) molecules [3], with consequent T-cell response and production of autoantibodies against type 2 transglutaminase (anti-tTG2-Ab). HLA phenotype is also considered the most important genetic marker of nonresponders to the hepatitis B (HBV) vaccination. In particular, the immune response to the HBV vaccine is largely determined by the presence of the immunogenetic peptides via the HLA-DR and DQ molecules [4][5], with the DR3-DQ2 and DR7-DQ2 haplotypes generally having a lower response rate [6][7][8][9].

In 2000, the World Health Organization estimated that 2 billion people worldwide had serological evidence of past or present infection with HBV and that 350 million of these people were chronically infected and at risk for HBV-related liver disease [10]. HCV infection is endemic to most parts of the world, although there is considerable geographic variation [11]. Estimates indicate that 2.2% of the global population is infected with HCV [12]. HCV is the most common chronic blood-borne infection in the U.S. and is a major cause of cirrhosis and hepatocellular carcinoma [13]. An interesting chapter in clinical practice is the possible association between CD and viral hepatitis. Recently, researchers have hypothesized that nonintestinal inflammatory chronic diseases, such as HBV and HCV, may be the immunologic trigger for the development of CD [14][15]. However, the association between chronic viral hepatitis and the development of CD is still a matter of debate. In a recent study, Leonardi and La Rosa found no cases of CD in a retrospective cohort of patients carrying HBV, and no CD cases appeared during treatment with interferon [16]. This study, although limited by the small size of patients studied, is interesting because it may be representative of what has been observed in Italy. HBV prevalence in Italy is higher than in the rest of Europe [17], and a high prevalence of CD is estimated as well [18]. A more interesting question is: if it does not seem to be an association between CD and viral hepatitis in Italy, what is happening in other regions of the world that have a high incidence of viral hepatitis [19][20][21] and an increased incidence of CD [22][23]? Until further, large, epidemiological studies investigate this question, the answer is still open.We have more answers about what happens in coeliacs after vaccination. Mass immunization of the population has been recommended by the World Health organization since 1991 [24], and it is generally performed in 12-year-old schoolchildren [25]. We know that CD patients have a lower rate of immunization after HBV vaccination [26][27]. We do not know if CD will increase with the actual rate of vaccination. This may be a problem for the public health system because a great deal of young people may be at high risk of contracting HBV due to a lack of immunization. In a fine paper published in 2008, Nemes et al. demonstrated that the response to HBV vaccination in CD patients is related to the response to a gluten-free diet [28]. In fact, an adequate vaccine response to HBV was found in coeliacs compliant with (Gluten-Free Diets) GFD, whereas nonresponse was a sign of undiagnosed CD or a lack compliance to GFD [28]. Surprisingly, Nemes et al. did not find any association between nonresponse and HLA-DQ2 or DQ8 status [28]. These results have been recently confirmed by Ertem et al., who found that response to HBV vaccine in children with CD who are compliant with GFD did not differ from the response in a healthy population [29]. Therefore, until new epidemiological data shed light on the possible association between CD and HBV infection, good advices seems to be

1. to screen for CD in schoolchildren before HBV vaccination;

2. to obtain optimal compliance to GFD in CD patients before HBV vaccination to reduce the risk of unresponsiveness;

3. to revaccinate during a well-controlled GFD in order to maintain a high level of immunization.
